# Editorial: Osteoporosis, sarcopenia and muscle-bone crosstalk in COPD

**DOI:** 10.3389/fphys.2022.1040693

**Published:** 2022-10-05

**Authors:** Yongchang Sun, Lijiao Zhang, Hua Cai, Yahong Chen

**Affiliations:** ^1^ Department of Respiratory and Critical Care Medicine, Peking University Third Hospital, and Research Center for Chronic Airway Diseases, Peking University Health Science Center, Beijing, China; ^2^ Department of Anesthesiology, Department of Medicine, David Geffen School of Medicine, University of California Los Angeles, Los Angeles, CA, United States

**Keywords:** COPD, comorbidity, sarcopenia, osteoporosis, muscle-bone crosstalk

Chronic obstructive pulmonary disease (COPD), characterized by persistent airflow limitation and respiratory symptoms, and mostly associated with cigarette smoking, is a prevalent chronic airway disease with high morbidity and mortality worldwide. COPD is further complicated by systemic diseases/comorbidities, such as skeletal muscle dysfunction/sarcopenia and osteoporosis, among others, which contribute significantly to poor outcomes of the disease.

A recent meta-analysis showed that the overall pooled prevalence of osteoporosis in patients with COPD was 38% (95%CI, 34%–43%), while lower BMI and sarcopenia were risk factors for osteoporosis in COPD (Chen et al., 2019), in addition to the use of glucocorticoids in these patients. The underlying mechanistic links between COPD and osteoporosis have received much attention, but still remain elusive, although there is evidence suggesting that systemic inflammation, presumably resulting from “overspill” of inflammatory mediators from the lungs (Sinden and Stockley, 2010), may play key roles, among other factors, in the pathogenesis of COPD-related osteoporosis. At the cellular level, bone loss mainly results from excessive resorption by osteoclasts, whose differentiation and activation are dependent on receptor activator of nuclear factor-κB ligand (RANKL). Our previous studies revealed higher level of RANKL in COPD, and in a recent study, we found that IL-17A contributed to bone loss induced by long-term cigarette smoke (CS) exposure in a well-established mouse model of COPD, probably through its proinflammatory effects and upregulation of RANKL expression in bone tissues (Xiong et al., 2020). These results suggest that COPD and its comorbidities may have common links, as IL-17 has also been implicated in the pathogenesis of lung diseases. In fact, an earlier study by Tsantikos et al. demonstrated that granulocyte colony-stimulating factor (G-CSF) might play a central and pathogenic role in COPD and its complex comorbidities including osteoporosis, loss of fat reserves and heart abnormalities (Tsantikos et al., 2018).

Similar to osteoporosis, sarcopenia affects a large number of COPD patients, with an overall pooled prevalence of 27.5% (95%CI, 18.4%–36.5%) (Sepúlveda-Loyola et al., 2020), while the prevalence increased with age, degree of airflow limitation and severity of disease (Jones et al., 2015). The confirmation of sarcopenia requires detection of low muscle quantity and quality, while poor physical performance is indicative of severe sarcopenia (Cruz-Jentoft et al., 2019). Ultrasonography is a noninvasive, nonradioactive tool used to measure muscle quantity. Earlier studies in patients with COPD demonstrated decreased size of the rectus femoris by grayscale ultrasound measurements, which were related to pulmonary function, fat-free mass, and physical performance (Seymour et al., 2009). However, grayscale ultrasonography only provides a measure of muscle quantity but not of muscle quality and function. Shear wave ultrasound elastography (SWE) is a potentially novel tool to evaluate qualitative muscle parameters. The study by Deng et al. investigated the feasibility of SWE to measure the stiffness of the rectus femoris and evaluated its value in predicting sarcopenia in patients with COPD. Compared with grayscale ultrasonography, SWE was not affected by the patient’s height, weight, or BMI and better represented skeletal muscle function and physical function, indicating that SWE is a potentially promising tool to predict sarcopenia in patients with COPD.

Considering that chest CT is widely used for lung assessment in COPD, concurrent measurement of the pectoris muscles has emerged as a modality for evaluation of sarcopenia in COPD. CT-derived pectoralis muscle area (PMA), a measure readily accessible in the clinical setting, provided relevant information of COPD morbidity (McDonald et al., 2014). The study by Qiao et al. further compared the densities and areas of the pectoralis muscles derived from chest CT scan between patients with COPD and healthy controls and explored whether the PMA and the pectoralis muscle attenuation (PMT) were associated with clinical indices of the patients. They found that PMA and PMT were lower in COPD patients, more significantly in those with acute exacerbation, and were associated with severity of emphysema and airway abnormalities. The study indicates that CT-derived measurements of the pectoralis muscle were valuable in detecting impaired muscle quantity and quality and predicting disease severity of COPD. In addition to PMA, attenuation measurement of the thoracic spine on routine chest CT may also provide useful information on bone heath in patients with COPD (Romme et al., 2012).

The underlying mechanisms linking COPD and comorbid sarcopenia have been investigated in recent years. Multiple factors have been proposed to contribute to muscle dysfunction in COPD, including inactivity, ageing, use of glucocorticoids, catabolic imbalance, nutritional changes, as well as systemic and local inflammation, oxidative stress, hypoxia and hypercapnia. These factors alone or in combination can lead to a reduction in muscle mass, deterioration of muscle bioenergy metabolism, defects in muscle repair and regeneration mechanisms, apoptosis and other anatomical and/or functional pathological changes, resulting in a decrease in the muscles’ ability to work (Ma K, et al). In another elegant and extensive review (Taivassalo T and Hepple RT), the authors provided convincing evidence showing that skeletal muscles of COPD patients exhibited an increased frequency of mitochondrial permeability transition events, while mitochondrial permeability transition was identified as a mechanism of muscle atrophy operating through mitochondrial reactive oxygen species (ROS) and caspase 3. It is also interesting to note that, mice chronically exposed to CS showed muscle atrophy, mitochondrial impairment, and neuromuscular junction alterations, even in the absence of significant lung disease, suggesting a role of long-term CS exposure in initiating the muscle disease in COPD. Taivassalo T and Hepple RT further analyzed the critical role of aryl hydrocarbon receptor (AHR) in driving muscle impairment, revealing novel pathways and potential targets for therapy of sarcopenia in COPD.

Sarcopenia and osteoporosis share common risk factors in COPD, including cigarette smoking, reduced level of physical activity, systemic inflammation, and oxidative stress. Furthermore, muscle and bone also serve as endocrine organs by producing myokines and osteokines to participate in reciprocal regulation, i.e. a mechanism of muscle-bone crosstalk. However, the implications of muscle-bone crosstalk in musculoskeletal comorbidity of COPD have been rarely investigated (Zhang and Sun, 2021). Recent studies have revealed the potential roles of a variety of myokines and osteokines, such as IL-6 (Chowdhury et al., 2020), fibronectin type III domain-containing protein 5 (FNDC5)/irisin (Zhang et al., 2022), myostatin (Severinsen and Pedersen, 2020), RANKL/RANK (Xiong et al., 2021) and osteocalcin (Chowdhury et al., 2020), in the development of sarcopenia and osteoporosis in COPD. Based on these findings, we propose a muscle-bone crosstalk mechanism in the pathogenesis of musculoskeletal comorbidity of COPD (Figure 1). Identification of key molecules in the delicate network of myokines and osteokines in the pathogenesis of musculoskeletal diseases holds promise for precision therapies. More clinically relevant is that, rehabilitation is an effective non-pharmacological therapy for improving outcomes of COPD, but its impact on the musculoskeletal comorbidity is underappreciated. Further understanding of muscle-bone crosstalk in physical exercise in COPD may provide evidence for novel modalities of non-pharmacological management for a disease currently without a pharmacological cure.

**FIGURE 1:**
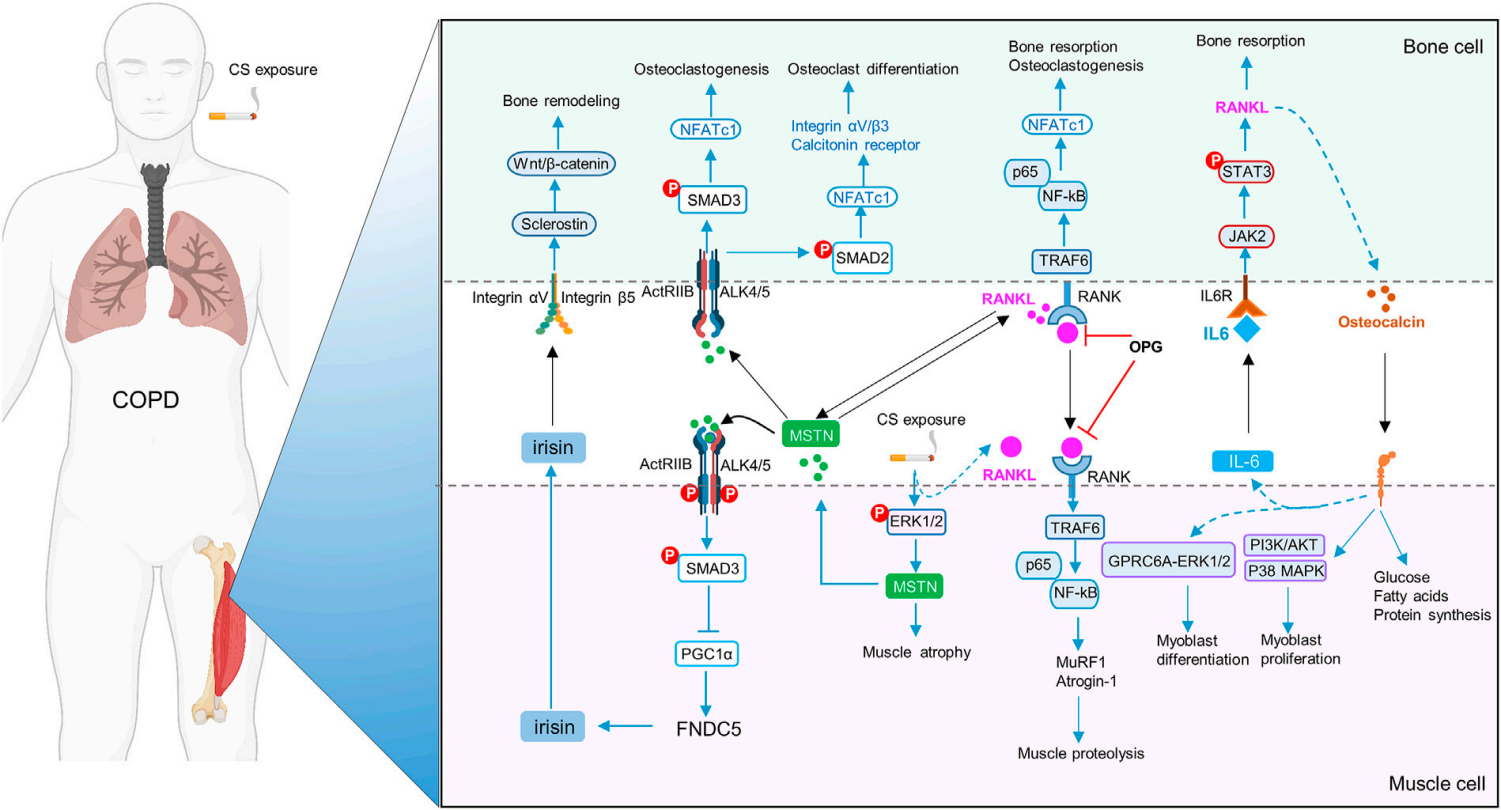
Schematic diagram of muscle-bone crosstalk in COPD. FNDC5/irisin is decreased, whereas IL-6, myostatin (MSTN) and RANKL/RANK are increased in skeletal muscles in COPD. Cigarette smoke extract (CSE), *via* enhanced p-ERK1/2, increases expression of MSTN, which further inhibits FNDC5/irisin expression through p-Smad3/PGC-1α pathway. MSTN also promotes osteoclastogenesis and osteoclast differentiation by NFATc1. Irisin upregulates sclerostin through integrin αV/β5 in the osteocytes, thereby inducing bone remodeling. RANKL activates the homologous receptor RANK on the surface of osteoclasts and osteoclast precursors, and activated RANK causes the recruitment of the adapter protein TRAF6, leading to NF-kB activation, thereby triggering the transcription of osteoclastogenic genes and bone resorption. Moreover, RANKL signaling can also lead to muscle dysfunction by upregulation of MSTN and activation of the ubiquitin-proteasome system (MuRF1 and Atrogin1) and the NF-κb pathway. OPG, the decoy receptor of RANKL, prevents osteoclast formation and bone loss. During exercise, muscle-derived IL-6 (mIL-6) acts on the IL-6 receptor of osteoblasts and increases the production of RANKL through STAT3 signaling pathway, subsequently promoting osteoclast differentiation and production of bioactive osteocalcin in osteoclasts. In turn, osteocalcin also enhances the production of mIL-6 during exercise, and promotes myoblast differentiation and proliferation.

## References

[B1] Chen Y. W. Ramsook A. H. Coxson H. O. Bon J. Reid W. D. (2019). Prevalence and risk factors for osteoporosis in individuals with COPD: A systematic review and meta-analysis. Chest 156, 1092–1110. 10.1016/j.chest.2019.06.036 31352034

[B2] Chowdhury S. Schulz L. Palmisano B. Singh P. Berger J. M. er al. (2020). Muscle-derived interleukin 6 increases exercise capacity by signaling in osteoblasts. J. Clin. Invest. 130, 2888–2902. 10.1172/JCI133572 32078586 PMC7260002

[B3] Cruz-Jientoft A. J. Bahat G. Bauer J. Boirie Y. Bruyere O. Cederholm T. (2019). Sarcopenia: Revised European consensus on definition and diagnosis. Age Ageing 48, 16–31. 10.1093/ageing/afy169 30312372 PMC6322506

[B4] Jones S. E. Maddocks M. Kon S. S. Canavan J. L. Nolan C. M. Clark A. L. (2015). Sarcopenia in COPD: Prevalence, clinical correlates and response to pulmonary rehabilitation. Thorax 70, 213–218. 10.1136/thoraxjnl-2014-206440 25561517

[B5] Mcdonald M. L. Diaz A. A. Ross J. C. Sanjoseestepar R. Zhou L. Regan E. A. (2014). Quantitative computed tomography measures of pectoralis muscle area and disease severity in chronic obstructive pulmonary disease. A cross-sectional study. Ann. Am. Thorac. Soc. 11, 326–334. 10.1513/AnnalsATS.201307-229OC 24558953 PMC4028743

[B6] Romme E. Murchison J. T. Phang K. F. Jansen F. H. Rutten E. Wouters E. (2012). Bone attenuation on routine chest CT correlates with bone mineral density on DXA in patients with COPD. J. Bone Min. Res. 27, 2338–2343. 10.1002/jbmr.1678 22692725

[B7] Sepulveda-Loyola W. Osadnik C. Phu S. Morita A. A. Duque G. Probst V. S. (2020). Diagnosis, prevalence, and clinical impact of sarcopenia in COPD: A systematic review and meta-analysis. J. Cachexia Sarcopenia Muscle 11, 1164–1176. 10.1002/jcsm.12600 32862514 PMC7567149

[B8] Severinsen M. C. K. Pedersen B. K. (2020). Muscle-organ crosstalk: The emerging roles of myokines. Endocr. Rev. 41, bnaa016. 10.1210/endrev/bnaa016 32393961 PMC7288608

[B9] Seymour J. M. Ward K. Sidhu P. S. Puthucheary Z. Steier J. Jolley C. J. (2009). Ultrasound measurement of rectus femoris cross-sectional area and the relationship with quadriceps strength in COPD. Thorax 64, 418–423. 10.1136/thx.2008.103986 19158125

[B10] Sinden N. Stockley R. A. (2010). Systemic inflammation and comorbidity in COPD: A result of 'overspill' of inflammatory mediators from the lungs? Review of the evidence. Thorax 65, 930–936. 10.1136/thx.2009.130260 20627907

[B11] Tsantikos E. Lau M. Castelino C. M. Maxwell M. J. Passey S. L. Hansen M. J. (2018). Granulocyte-CSF links destructive inflammation and comorbidities in obstructive lung disease. J. Clin. Invest. 128, 2406–2418. 10.1172/JCI98224 29708507 PMC5983324

[B12] Xiong J. Le Y. Rao Y. Zhou L. Hu Y. Guo S. (2021). RANKL mediates muscle atrophy and dysfunction in a cigarette smoke-induced model of chronic obstructive pulmonary disease. Am. J. Respir. Cell Mol. Biol. 64, 617–628. 10.1165/rcmb.2020-0449OC 33689672

[B13] Xiong J. Tian J. Zhou L. Le Y. Sun Y. (2020). Interleukin-17A deficiency attenuated emphysema and bone loss in mice exposed to cigarette smoke. Int. J. Chron. Obstruct. Pulmon. Dis. 15, 301–310. 10.2147/COPD.S235384 32103929 PMC7020917

[B14] Zhang L. Li C. Xiong J. Chang C. Sun Y. (2022). Dysregulated myokines and signaling pathways in skeletal muscle dysfunction in a cigarette smoke–induced model of chronic obstructive pulmonary disease. Front. Physiol. 13, 929926. 10.3389/fphys.2022.929926 36091368 PMC9454092

[B15] Zhang L. Sun Y. (2021). Muscle-bone crosstalk in chronic obstructive pulmonary disease. Front. Endocrinol. 12, 724911. 10.3389/fendo.2021.724911 PMC8505811 34650518

